# Characterization of the Mechanical Strength, Resorption Properties, and Histologic Characteristics of a Fully Absorbable Material (Poly-4-hydroxybutyrate—PHASIX Mesh) in a Porcine Model of Hernia Repair

**DOI:** 10.1155/2013/238067

**Published:** 2013-05-28

**Authors:** Corey R. Deeken, Brent D. Matthews

**Affiliations:** Department of Surgery, Section of Minimally Invasive Surgery, Washington University School of Medicine, St. Louis, MO 63110, USA

## Abstract

*Purpose*. Poly-4-hydroxybutyrate (P4HB) is a naturally derived, absorbable polymer. P4HB has been manufactured into PHASIX Mesh and P4HB Plug designs for soft tissue repair. The objective of this study was to evaluate mechanical strength, resorption properties, and histologic characteristics in a porcine model. *Methods*. Bilateral defects were created in the abdominal wall of *n* = 20 Yucatan minipigs and repaired in a bridged fashion with PHASIX Mesh or P4HB Plug fixated with SorbaFix or permanent suture, respectively. Mechanical strength, resorption properties, and histologic characteristics were evaluated at 6, 12, 26, and 52 weeks (*n* = 5 each). *Results*. PHASIX Mesh and P4HB Plug repairs exhibited similar burst strength, stiffness, and molecular weight at all time points, with no significant differences detected between the two devices (*P* > 0.05). PHASIX Mesh and P4HB Plug repairs also demonstrated significantly greater burst strength and stiffness than native abdominal wall at all time points (*P* < 0.05), and material resorption increased significantly over time (*P* < 0.001). Inflammatory infiltrates were mononuclear, and both devices exhibited mild to moderate granulation tissue/vascularization. *Conclusions*. PHASIX Mesh and P4HB Plug demonstrated significant mechanical strength compared to native abdominal wall, despite significant material resorption over time. Histological assessment revealed a comparable mild inflammatory response and mild to moderate granulation tissue/vascularization.

## 1. Introduction

Biological scaffold materials derived from dermis, pericardium, and small intestine submucosa of human, bovine, and porcine origin have been utilized over the last decade for soft tissue repair applications such as hernia repair [1], breast reconstruction [2], staple-line reinforcement [3], and orthopedic applications [4]. These scaffold materials are particularly useful in clean-contaminated or contaminated settings due to their rapid revascularization and clearance of bacteria [5, 6]. Permanent, synthetic polymer mesh materials are not typically utilized in these settings due to risk of infection [7]. Biological scaffolds are also utilized as an alternative to fascial closure when there is excessive tension on the wound, when tissue loss makes closure especially difficult, or in “damage-control”/abdominal compartment settings in which the abdomen must be left open until the patient is stabilized [8]. In these settings, scaffolds are utilized to protect the abdominal contents, typically until granulation of the wound occurs and a split-thickness skin graft can be applied. Although biological scaffolds present many attractive advantages, these materials are also extremely expensive, exhibit substantial scaffold variability, and provoke patient-specific immunological responses. Thus, absorbable polymer scaffolds have recently been developed (Table 1). Early designs of absorbable polymer scaffolds included materials such as DEXON (Covidien, Mansfield, MA) and VICRYL (Ethicon, Inc., Somerville, NJ).

According to the Instructions for Use, DEXON is comprised of polyglycolic acid (PGA), and it degrades *in vivo* via hydrolysis. Full resorption of the PGA is expected to be complete in approximately 60–90 days (2-3 months). Animal studies have shown that DEXON is quickly resorbed and associated with hernia formation after just 14 days due to central mesh failure [9]. By 90 days, greater than 80% of the scaffolds were fully resorbed in that particular animal study [9]. Clinical studies have shown that DEXON has primarily been utilized in damage control situations to cover the open abdomen until granulation tissue is present and a skin graft can be applied [10]. However, subsequent development of a ventral hernia has also been observed in these patients. In one study, 6 of 8 (75%) patients developed a hernia after DEXON placement [11]. The authors concluded that the DEXON mesh was useful for providing a temporary support for the abdominal wall but would likely require a ventral hernia repair with placement of a permanent mesh once the contamination was resolved, making this material a less than ideal long-term solution.

VICRYL is a copolymer of glycolide and lactide which also degrades *in vivo* through hydrolysis. According to its Instructions for Use, VICRYL loses 77% of its strength in the first 14 days (0.5 months) in a rat model, and it is fully resorbed in approximately 60–90 days (2-3 months). Clinically, VICRYL has been utilized primarily in damage-control applications as a buttress to the abdominal wall [12] or as a prophylactic measure to prevent incisional hernia formation [13]. Animal studies have shown that the tensile strength of the rat abdominal wall at 30 and 60 days is equivalent whether VICRYL mesh or biological scaffolds were utilized (Surgisis and AlloDerm) [14]. However, collagen deposition and neovascularization were greater for the biological scaffolds than for VICRYL [14]. This could be explained by the decreased pH and associated increase in inflammation observed at the wound site as the VICRYL mesh degrades [14]. Pronounced inflammation, reduced tissue ingrowth, and greater angiogenesis were observed for VICRYL mesh compared to permanent PROLENE and ULTRAPRO meshes in another animal study involving a hamster model [15]. The authors of that study concluded that the more aggressive foreign body response incited by the resorbable VICRYL mesh did not lead to better tissue incorporation as predicted [15]. In a similar study, tissue ingrowth was also unsatisfactory for the absorbable VICRYL mesh when compared to permanent synthetic materials such as polypropylene and expanded polytetrafluoroethylene in a rabbit model [16].

Overall, both DEXON and VICRYL scaffolds lose mechanical strength and are resorbed fairly quickly, making them less than ideal for hernia repair applications which require more long-term support of the repair site until tissue remodeling is complete. Thus, more recent absorbable scaffold designs have been developed which utilize long-lasting polymers that degrade more slowly. Scaffolds such as GORE BIO-A (W.L. Gore and Associates, Inc., Flagstaff, AZ), TIGR Matrix (Novus Scientific, Uppsala, Sweden), and PHASIX Mesh (C. R. Bard, Inc./Davol Inc., Warwick, RI) fall into this category.

GORE BIO-A is a copolymer of poly(glycolide: trimethylene carbonate) that degrades *in vivo* through both hydrolytic and enzymatic mechanisms and is fully resorbed within approximately 180 days (6 months) according to the Instructions for Use. In a rat model with methicillin-resistant *Staphylococcus aureus* (MRSA) contamination, bacteria were cleared from the GORE BIO-A mesh more effectively than either VICRYL or TIGR Matrix at the 10^6^ inoculum [17]. However, at the 10^4^ inoculum, all three scaffolds performed equally. Overall, all three scaffolds exhibited reduced tensile strength and increased rate of mesh failure regardless of scaffold composition [17]. GORE BIO-A has also been utilized in a number of clinical applications including Amyand hernia repair [18], open elective hernia repair [19], paraesophageal/hiatal hernia repair [20], suture line reinforcement [20], pelvic floor reinforcement [20], and breast reconstruction [20]. The outcomes thus far have been promising with low rates of recurrence, infection, and pain. However, the majority of these studies have been case reports or very small series (i.e., less than 10 patients). Larger, more comprehensive clinical trials are needed to fully understand the long-term capabilities of this scaffold.

TIGR Matrix is knitted from two fibers having different resorption rates. According to the Instructions for Use, the first fiber makes up approximately 40% of the overall mesh by weight and is a copolymer of polyglycolide, polylactide, and polytrimethylene carbonate. This fiber degrades *in vivo* through hydrolysis, loses substantial mechanical strength in the first 14 days (0.5 months), and is fully resorbed in approximately 120 days (4 months). The second fiber makes up approximately 60% of the overall mesh by weight and is a copolymer of polylactide and polytrimethylene carbonate. This fiber also degrades *in vivo* through hydrolysis, but it retains its mechanical strength longer than the first fiber. It begins to demonstrate loss of mechanical strength after approximately 270 days (9 months) and is fully resorbed in approximately 1095 days (36 months). TIGR Matrix has been evaluated in a long-term animal model, and a clinical trial is currently underway. In the animal study, TIGR Matrix was compared to permanent polypropylene mesh in sheep with full thickness abdominal wall defects over the course of 4, 9, 15, 24, and 36 months [21]. The results showed a typical long-term inflammatory response to the permanent polypropylene contrasted with a gradual resorption of the TIGR Matrix until it was fully resorbed at 36 months [21]. The TIGR Matrix also exhibited collagen deposition at the repair site that increased over time and eventually resembled native connective tissue [21]. In the clinical trial, forty subjects were enrolled (*n* = 40) and followed for 1 year after placement of TIGR Matrix to repair a primary inguinal hernia [22]. Pain and recurrence were evaluated at 0.5, 1, 3, 6, and 12 months, and pain scores were reduced from an average of 17.4 before surgery to 0.3 after just 6 months [22].

PHASIX Mesh and P4HB Plug designs are both fabricated from poly-4-hydroxybutyrate (P4HB). P4HB is a natural polymer from the class of polyhydroxyalkanoates [23]. In nature, these polymers are produced by microorganisms for the purpose of regulating energy metabolism [23]. In the case of PHASIX Mesh, P4HB is a naturally derived, fully absorbable polymer produced by *Escherichia coli* K12 bacteria via transgenic fermentation techniques [23]. P4HB has a chemical structure very similar to many of the synthetic polyester polymers, but because it is biologically derived rather than chemically synthesized, P4HB does not contain any residues from metal catalysts that are typically utilized during chemical synthesis of other polyester polymers [23]. P4HB degrades *in vivo* through both hydrolysis and a hydrolytic enzymatic digestive process and is fully resorbed in approximately 365–545 days (12–18 months) according to the Instructions for Use. The resulting by-products (carbon dioxide and water) are metabolized very quickly via the Krebs Cycle and beta-oxidation [23]. Unlike absorbable scaffolds such as DEXON and VICRYL, whose by-products decrease the pH at the wound site, degradation of P4HB is not as acidic, which may reduce the inflammatory response associated with these materials [23]. In addition, P4HB has been shown to degrade more slowly than PGA, yielding more gradual loss of mechanical strength [23]. This is advantageous in applications such as hernia repair in which the rate of degradation should ideally match the rate of remodeling and neotissue deposition at the repair site. A gradual change in mechanical properties is also advantageous because it leads to a gradual transfer of the load from the scaffold back to the tissue, which may help to prevent hernia recurrence. P4HB has been evaluated in a number of animal studies over the past decade, particularly those investigating cardiovascular applications such as tissue engineered trileaflet heart valves [24], artery augmentation patches [25], and small diameter vascular grafts [26], as well as development of P4HB as a suture material [27]. However, this is the first study to evaluate this material specifically for hernia repair applications. Thus, the purpose of this study was to determine the mechanical properties, resorption profile, and histological characteristics of PHASIX Mesh and P4HB Plug compared to the native abdominal wall when utilized to bridge a surgical defect in a porcine model. 

## 2. Materials and Methods

### 2.1. Materials

**Figure 1:**
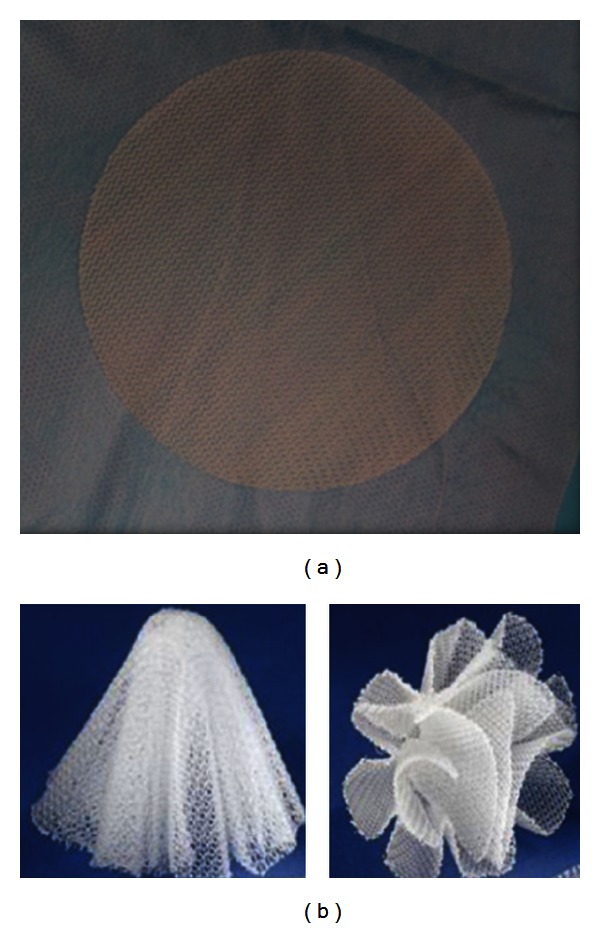
(a) PHASIX Mesh comprised of a fully resorbable polymer (poly-4-hydroxybutyrate, P4HB) monofilament knitted into a flat sheet configuration. (b) P4HB Plug comprised of a fully resorbable polymer (poly-4-hydroxybutyrate, P4HB) monofilament preformed into a three-dimensional shape with a fluted outer layer and inner layers or ‘‘petals” attached at the tip.

PHASIX Mesh is comprised of a fully resorbable polymer monofilament (poly-4-hydroxybutyrate, P4HB) that is knitted into a flat sheet configuration as shown in Figure 1(a). The P4HB Plug design is also comprised of P4HB monofilament, but it is preformed into a three-dimensional shape with a fluted outer layer and inner layers or “petals” attached at the tip as shown in Figure 1(b).

### 2.2. Preimplantation Characterization

#### 2.2.1. Suture Retention Strength Testing

Twelve specimens (*n* = 12) measuring 2.5 × 5.1 cm (1 × 2 in) were prepared from PHASIX Mesh and subjected to suture retention testing at time zero, *T*_0_ (i.e., prior to implantation). Six of the specimens (*n* = 6) were oriented such that the load applied during testing was parallel to the longest dimension of the mesh interstices. The other six (*n* = 6) specimens were oriented such that the load applied during testing was perpendicular to the longest dimension of the mesh interstices. A custom test fixture was utilized in which the mesh specimen was loaded vertically in the Instron machine (Instron, Norwood, MA) with a gauge length of 2.5 cm (1 in) and clamped along the upper edge using pneumatic grips set to 60 psi. A stainless steel wire with a diameter of 0.36 mm (simulating 0 polypropylene suture material) was passed through the mesh 1.0 cm from the bottom edge of the mesh. This was done to capture at least two rows of mesh interstices. Each specimen was tested in tension at a rate of 300 mm/min (12 in/min) until the suture pulled through the mesh. The “suture retention strength” was recorded as the maximum load sustained by the mesh in units of Newtons (N).

#### 2.2.2. Tear Resistance Testing

Tear resistance testing was based on the American Society for Testing and Materials (ASTM) specification #D2261-07a. For this type of testing, twelve mesh specimens (*n* = 12) measuring 2.5 × 7.6 cm (1 × 3 in) were prepared from PHASIX Mesh and subjected to tear resistance testing at time zero, *T*_0_ (i.e., prior to implantation). A 2.5 cm (1 in) slit was cut from the center of the 2.5 cm edge of the specimen toward the center of the mesh to form two tabs. The left tab was clamped in the upper grip of the Instron machine using a pneumatic grip set to 60 psi, and the right tab was clamped in an identical fashion in the lower grip. This arrangement yielded a 2.5 cm gauge length (1 in). Six of the specimens (*n* = 6) were oriented such that the load applied during testing was parallel to the longest dimension of the mesh interstices. Conversely, the other six (*n* = 6) specimens were oriented such that the load applied during testing was perpendicular to the longest dimension of the mesh interstices. The test was conducted in tension at a rate of 300 mm/min (12 in/min) until the specimen tore in half. The “tear strength” was recorded as the maximum load sustained by the mesh in units of Newtons (N).

#### 2.2.3. Ball Burst Testing

Six circular specimens (*n* = 6) measuring 7.5 cm in diameter (3 in diameter) were prepared from PHASIX Mesh and subjected to ball burst testing at time zero, *T*_0_ (i.e., prior to implantation). Mesh orientation was not considered due to the biaxial nature of the test. For this reason, only one set of mesh specimens was prepared with no indication of the orientation of the mesh interstices during testing. A custom test fixture was fabricated based on ASTM specification #D3787-07. Two circular stainless steel rings were utilized to clamp the mesh specimen to prevent slipping during the test. Then, a 2.5 cm diameter (1 in) stainless steel ball was applied in compression at a rate of 300 mm/min (12 in/min) until it burst through the mesh. The ultimate tensile stress and the strain at a stress of 16 N/cm were recorded in units of N/cm and percent, respectively. 

### 2.3. Animal Model

#### 2.3.1. Study Compliance

The Institutional Animal Care and Use Committee (IACUC) of the CBSET, Inc. facility (Lexington, MA) where the study was conducted approved the experimental protocol prior to the start of the study, and standard operating procedures were followed at all times.

#### 2.3.2. Surgical Technique

**Figure 2:**
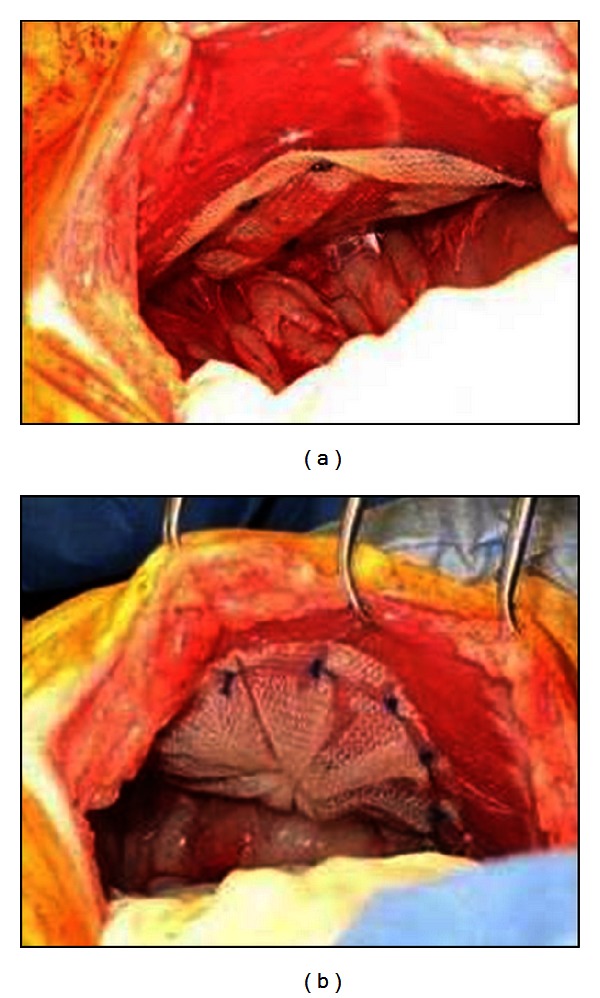
(a) PHASIX Mesh placed in the preperitoneal plane (bridging the defect) and fixated circumferentially with approximately *n* = 16 SorbaFix Absorbable Fixation Device constructs. (b) P4HB Plug placed in the preperitoneal plane as an onlay mesh bridging the defect and fixated circumferentially with approximately *n* = 12 permanent PROLENE (2-0) sutures.

Twenty (*n* = 20) castrated male, Yucatan minipigs weighing 33.7–41.8 kg (at surgery) were acquired for the study and acclimated to the facility for a minimum of 21 days. Animals were fasted for at least 12 hours prior to surgery. On the morning of surgery, Buprenorphine (0.03 mg/kg, IM) and Rimadyl (2.2 mg/kg, PO) were administered. Following sedation, the animals were intubated and maintained under anesthesia with 0.5–5%, isoflurane inhalant anesthetic, to effect. The animals were placed in dorsal recumbency, and the ventral abdomen was prepared for aseptic survival surgery by shaving the entire abdominal region, cleaning the operative area with three alternating scrubs of povidone-iodine solution and 70% isopropyl alcohol solution, and applying sterilized surgical drapes over the entire field. Following preparation of the abdomen, a midline laparotomy (~30 cm) was performed. Two, 3 cm diameter (1.2 in diameter) bilateral muscular defects were created in the anterior abdominal wall using a preperitoneal approach (i.e., the peritoneum remained intact). The surgical defects were not closed and were bridged with a 10.2 cm diameter (4 in diameter) PHASIX Mesh on the right side (Figure 2(a)) or a 7.9 cm diameter (3.1 in diameter) P4HB Plug on the left side (Figure 2(b)). The PHASIX Mesh was placed in the preperitoneal plane (bridging the defect) and fixated circumferentially with approximately *n* = 16 SorbaFix Absorbable Fixation Device constructs comprised of poly(D,L-lactide). The P4HB Plug was placed in the preperitoneal plane as an onlay mesh bridging the defect and fixated circumferentially with approximately *n* = 12 permanent PROLENE (2-0) sutures. The abdominal midline was then repaired via standard closure techniques, and the skin was tattooed to define the cranial, caudal, medial, and lateral aspects of each device to facilitate identification at the time of explant. The animals were recovered from anesthesia and allowed free access to food and water *ad libitum*. Buprenorphine (0.02 mg/kg, IM) was administered every 4–12 hours, and Rimadyl (2.2 mg/kg, PO) was administered every 24 hours for 72 hours postoperatively.

The abdominal region of each animal was examined daily to assess the condition of the wound and the subcutaneous tissues for evidence of herniation, diastasis, seromas, and/or hematomas. Animals were survived for 6, 12, 26, or 52 weeks (*n* = 5 in each group), followed by euthanasia. Humane euthanasia was carried out after sedation with Telazol (4 mg/kg, IM) and anesthesia via inhalant isoflurane. Euthanasia was achieved by administering an overdose of sodium pentobarbital (60–150 mg/kg, intravenously, to effect) in accordance with the American Veterinary Medical Association (AVMA) Panel of Euthanasia and Journal of the American Veterinary Medical Association. Following euthanasia, the abdominal skin was dissected from the entire abdomen, and the abdominal wall (including the 2 surgical defects) was excised, photographed, and placed in saline solution (0.9% NaCl) for subsequent mechanical testing. A representative specimen from each PHASIX Mesh and P4HB Plug repair was also harvested for histological and molecular weight analyses. This specimen was split into two pieces. One half was placed in 10% neutral buffered formalin for histological analysis, and the other was placed in an empty specimen jar for molecular weight analysis.

### 2.4. Postimplantation Characterization

The repair sites (i.e., abdominal wall tissue plus test device as a composite specimen) were subjected to ball burst testing to evaluate the peak load and relative stiffness of each repair site compared to native abdominal wall. The peritoneum was carefully removed from both of the repair sites and the native abdominal wall sites prior to testing to eliminate the contribution of the peritoneum and to allow assessment of the strength of the repair site alone.

#### 2.4.1. Mechanical Testing

The repair sites and native abdominal wall sites were subjected to burst testing using an Instron servohydraulic test frame (Instron, Norwood, MA). A 0.95 cm (0.375 in) diameter ball was applied in compression at a rate of 25.4 mm/min (1 inch/min) until the ball burst through. The peak load (ball burst force in units of Newtons, N) sustained by each specimen was recorded, and the relative stiffness was calculated from the slope of the line formed when load was graphed versus elongation (units = N/mm). Relative stiffness was taken from the slope of the line in the region of 30–80% of the peak load.

#### 2.4.2. Gel Permeation Chromatography (GPC)

Explanted PHASIX Mesh and P4HB Plug specimens were enzymatically digested and manually cleaned to remove residual tissue prior to gel permeation chromatography (GPC) analysis. GPC was conducted to quantify the molecular weight of the mesh material remaining at each time point. Specimens were placed in 50 mL tubes containing 40 mL collagenase (type I) solution (1.0 mg/mL) in TESCA buffer (50 mM TES, 2 mM CaCl_2_, 10 mM NaN_3_, pH 7.4). The tubes were placed on a shaker (50 rpm) and incubated at 37°C to digest the tissue. After overnight incubation (~17 hours), the specimens were removed from the buffer and manually cleaned of tissue. The cleaned specimens were rinsed in distilled water, followed by 70% ethanol, and dried prior to GPC analysis. Cleaned mesh specimens were dissolved at 1 mg/mL in chloroform, filtered using a 0.45 *μ*m filter to remove undigested particulates, and 95 *μ*L of this solution was then injected onto a GPC column for analysis. GPC was performed in chloroform at 1 ml/min using a Polymer Labs, PLgel column (5 micron, mixed C, 300 × 7.5 mm) with an Agilent 1100 Series HPLC with RI detector. Calibration was conducted against monodisperse polystyrene standards. Molecular weight is reported below in units of Daltons (Da).

#### 2.4.3. Histology

All histological assessments were conducted by a board-certified veterinary pathologist (CBSET, Lexington, MA). The explanted specimens were immersion-fixed in 10% neutral buffered formalin, cut into cross-section, and paraffin-embedded for further processing. Paraffin-embedded sections were microtomed (4-5 *μ*m), mounted onto glass slides, and stained with hematoxylin and eosin (H&E), Masson's Trichrome, and Picrosirius Red to characterize the host inflammatory/fibrotic response, collagen deposition/remodeling, and absorption properties associated with each specimen based on a standardized scoring system. Specimens were scored for inflammatory cell infiltrates (neutrophils, eosinophils, macrophages, lymphocytes, giant cells), neovascularization, fibroplasia (granulation tissue), hemorrhage, necrosis, and fibrosis using the following scoring system as described previously [28]:

 0 = absent/no response
 1 = minimal/barely detectable
 2 = mild/slightly detectable
 3 = moderate/easily detectable
 4 = marked/very evident.

Picrosirius Red stained slides were viewed via cross-polarization microscopy. Newly deposited, type III collagen appeared green, while mature, type I collagen appeared red or orange under these conditions. A score of “1” was assigned when green (type III collagen) predominated, a score of “2” was assigned when there was a mixture of green, yellow, and yellow-orange, and a score of “3” was assigned when red-orange (type I collagen) predominated.

#### 2.4.4. Statistical Analysis

Systat software (version 12.0, Systat Software, Inc., San Jose, CA) was utilized to perform all statistical analyses. For continuous data in which three or more groups of data were compared, (i.e., peak burst load, relative stiffness, molecular weight) a one-way analysis of variance (ANOVA) was performed followed by a Fisher's LSD post-test as appropriate. For scores such as histological parameters in which three or more groups of data were compared, a nonparametric test (Kruskal-Wallis) was performed followed by a Dunn's posttest as appropriate. Statistical significance was set at the *P* < 0.05 level. All data are reported as mean ± standard error of the mean (SEM) except the histological parameters in which the median is reported.

## 3. Results

### 3.1. Preimplantation Characterization

#### 3.1.1. Suture Retention Strength Testing

When PHASIX Mesh was evaluated in the direction *parallel* to the longest dimension of the interstices, the suture retention strength was 59.16  ±  5.7 N, which was greater than the 20 N suture retention strength suggested for hernia repair applications [29, 30]. Similarly, when tested in the direction *perpendicular* to the longest dimension of the interstices, the suture retention strength of PHASIX Mesh was again greater than 20 N at 49.10 ± 2.3 N.

#### 3.1.2. Tear Resistance Testing

When PHASIX Mesh was evaluated in the direction *parallel* to the longest dimension of the interstices, the tear resistance strength was 30.33 ± 3.1 N, which was greater than the 20 N tear resistance strength suggested for hernia repair applications [29, 30]. Similarly, when tested in the direction *perpendicular* to the longest dimension of the interstices, the tear resistance strength of PHASIX Mesh was again greater than 20 N at 29.48 ± 2.4 N.

#### 3.1.3. Ball Burst Testing

When subjected to ball burst testing, the maximum compressive load sustained by the PHASIX Mesh was 486.97 ± 12.6 N, with a tensile strength of 140.70 ± 5.4 N/cm and a strain at a stress of 16 N/cm of 15.43 ± 0.2%. Tensile strength greater than 50 N/cm and strain in the range of 10–30% are considered suitable properties for hernia repair applications [29, 30]. Thus, PHASIX Mesh possessed appropriate tensile strength and strain values at time zero, *T*_0_ (prior to implantation).

### 3.2. Postimplantation Characterization

#### 3.2.1. Observations during Survival Period

One animal died prior to its expected time point for reasons unrelated to the materials being evaluated in this study (i.e., sedation-related complication). This animal was replaced with an additional animal in order to maintain *n* = 5 animals in each group. All other animals survived until their expected time points.

#### 3.2.2. Observations during Explantation of Devices

**Figure 3:**
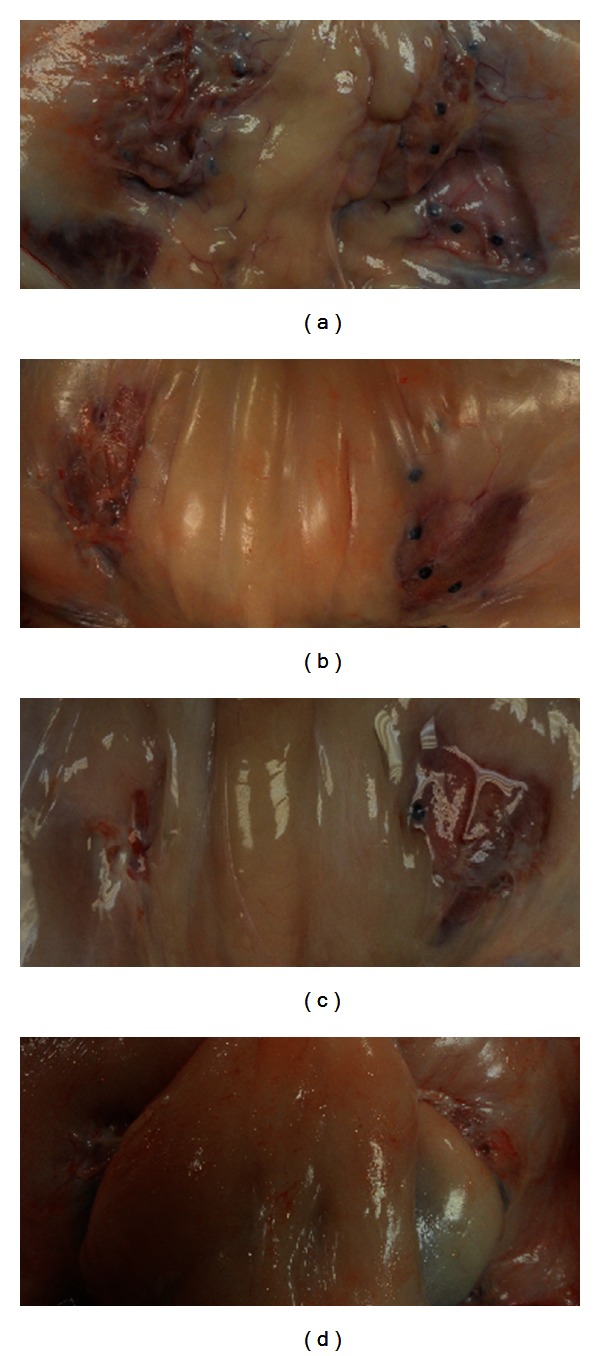
Macroscopic appearance of PHASIX Mesh (right side) and P4HB Plug (left side) at (a) 6 weeks, (b) 12 weeks, (c) 26 weeks, and (d) 52 weeks. An increase in preperitoneal adipose tissue was observed over time, with no evidence of hernia and/or diastasis.

As shown in Figures 3(a)–3(d) both devices (i.e., PHASIX Mesh and P4HB Plug) and both types of fixation (i.e., SorbaFix Absorbable Fixation Device fasteners and PROLENE sutures) remained visible and intact at the repair sites at 6, 12, 26, and 52 weeks. No evidence of hernia or diastasis was documented in any of the animals at any of the time points.

#### 3.2.3. Burst Strength (N)

**Figure 4:**
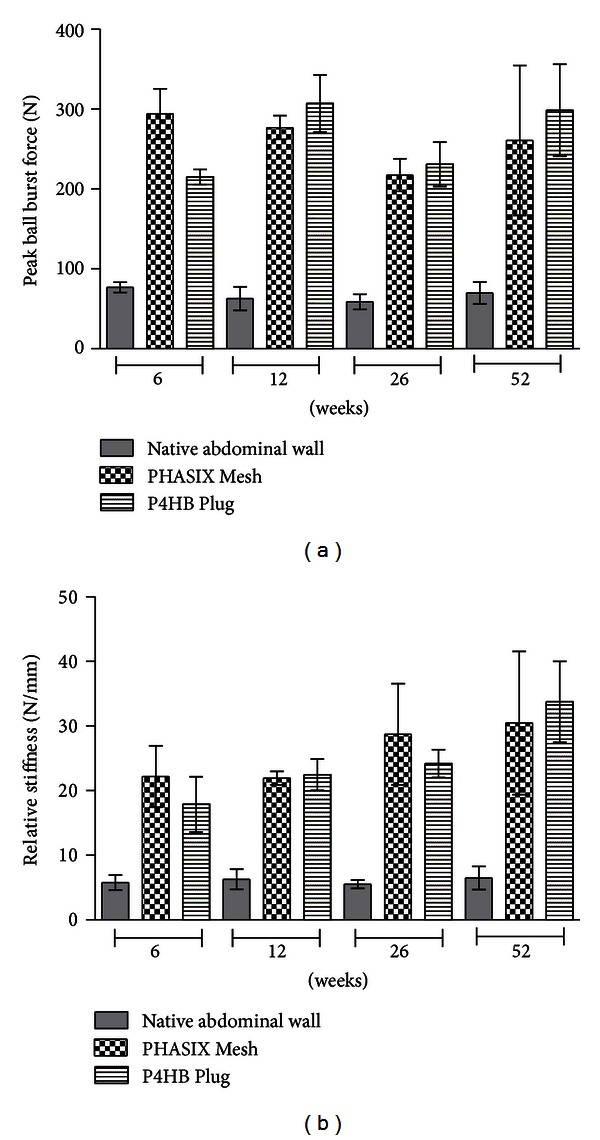
Mechanical properties of the native abdominal wall compared to PHASIX Mesh and P4HB Plug at 6, 12, 26, and 52 weeks: (a) peak load (N) and (b) relative stiffness (N/mm).

As shown in Figure 4(a), the burst strength of the native abdominal wall tissue remained stable throughout the duration of the study with no significant changes over time. At 6, 12, 26, and 52 weeks, the burst strength of the native abdominal wall was 76.9 ± 6.3 N, 62.8 ± 14.7 N, 58.7 ± 9.4 N, and 69.7 ± 13.6 N, respectively. No significant differences were detected between 6 and 12 weeks (*P* = 0.788), between 12 and 26 weeks (*P* = 0.938), between 26 and 52 weeks (*P* = 0.834), or overall between 6 and 52 weeks (*P* = 0.891).

The burst strength of the abdominal wall repaired with PHASIX Mesh also remained stable throughout the duration of the study with no significant changes over time. At 6, 12, 26, and 52 weeks, the burst strength of the PHASIX Mesh repair site was 294.0 ± 31.4 N, 277.0 ± 14.8 N, 217.6 ± 20.3 N, and 260.7 ± 93.8 N, respectively. No significant differences were detected between 6 and 12 weeks (*P* = 0.746), between 12 and 26 weeks (*P* = 0.262), between 26 and 52 weeks (*P* = 0.414), or overall between 6 and 52 weeks (*P* = 0.527).

The burst strength of the abdominal wall repaired with P4HB Plug also remained stable throughout the duration of the study with no significant changes over time. At 6, 12, 26, and 52 weeks, the burst strength of the P4HB Plug repair site was 215.2 ± 9.3 N, 307.0 ± 36.0 N, 231.0 ± 28.0 N, and 298.5 ± 57.6 N, respectively. No significant differences were detected between 6 and 12 weeks (*P* = 0.086), between 12 and 26 weeks (*P* = 0.153), between 26 and 52 weeks (*P* = 0.204), or overall between 6 and 52 weeks (*P* = 0.118).

PHASIX Mesh and P4HB Plug repairs demonstrated similar burst strengths at 6, 12, 26, and 52 weeks with no significant differences detected between the two devices at any of the time points evaluated (*P* = 0.139, *P* = 0.568, *P* = 0.798, and *P* = 0.474, resp.). In addition, porcine abdominal wall sites repaired with PHASIX Mesh or P4HB Plug materials both demonstrated significantly greater burst strength compared to the native abdominal wall at 6, 12, 26, and 52 weeks regardless of whether the mesh (*P* < 0.001, *P* < 0.001, *P* = 0.004, and *P* = 0.001, resp.) or the plug (*P* = 0.011, *P* < 0.001, *P* = 0.002, and *P* < 0.001, resp.) was utilized to bridge the defect. 

#### 3.2.4. Relative Stiffness (N/mm)

As shown in Figure 4(b), the relative stiffness of the native abdominal wall tissue remained stable throughout the duration of the study with no significant changes over time. At 6, 12, 26, and 52 weeks, the relative stiffness of the native abdominal wall was 5.8 ± 1.2 N/mm, 6.3 ± 1.6 N/mm, 5.5 ± 0.7 N/mm, and 6.5 ± 1.8 N/mm, respectively. No significant differences were detected between 6 and 12 weeks (*P* = 0.940), between 12 and 26 weeks (*P* = 0.910), between 26 and 52 weeks (*P* = 0.887), or overall between 6 and 52 weeks (*P* = 0.917).

The relative stiffness of the abdominal wall repaired with PHASIX Mesh also remained stable throughout the duration of the study with no significant changes over time. At 6, 12, 26, and 52 weeks, the relative stiffness of the PHASIX Mesh repair site was 22.2 ± 4.7 N/mm, 21.9 ± 1.0 N/mm, 28.7 ± 7.8 N/mm, and 30.5 ± 11.1 N/mm, respectively. No significant differences were detected between 6 and 12 weeks (*P* = 0.970), between 12 and 26 weeks (*P* = 0.329), between 26 and 52 weeks (*P* = 0.799), or overall between 6 and 52 weeks (*P* = 0.234).

The relative stiffness of the abdominal wall repaired with P4HB Plug also remained stable throughout the duration of the study with no significant changes over time. At 6, 12, 26, and 52 weeks, the relative stiffness of the P4HB Plug repair site was 17.9 ± 4.3 N/mm, 22.5 ± 2.4 N/mm, 24.2 ± 2.2 N/mm, and 33.8 ± 6.3 N/mm, respectively. No significant differences were detected between 6 and 12 weeks (*P* = 0.508), between 12 and 26 weeks (*P* = 0.804), or between 26 and 52 weeks (*P* = 0.170). However, there was a slight trend toward greater stiffness overall at 52 weeks compared to 6 weeks (*P* = 0.025).

PHASIX Mesh and P4HB Plug repairs demonstrated similar relative stiffness at 6, 12, 26, and 52 weeks with no significant differences detected between the two devices at any of the time points evaluated (*P* = 0.533, *P* = 0.940, *P* = 0.512, and *P* = 0.635, resp.). In addition, porcine abdominal wall sites repaired with PHASIX Mesh or P4HB Plug materials both demonstrated significantly greater relative stiffness compared to the native abdominal wall at 6, 12, 26, and 52 weeks regardless of whether the mesh (*P* = 0.021, *P* = 0.027, *P* = 0.001, and *P* = 0.001, resp.) or the plug (*P* = 0.084, *P* = 0.023, *P* = 0.009, and *P* < 0.001, resp.) was utilized to bridge the defect. As demonstrated by a *P* value of 0.084, the stiffness of the abdominal wall sites repaired with P4HB Plug materials at 6 weeks did not quite reach statistical significance and were only trending toward greater relative stiffness compared to the native abdominal wall.

#### 3.2.5. Molecular Weight (Da)

**Figure 5:**
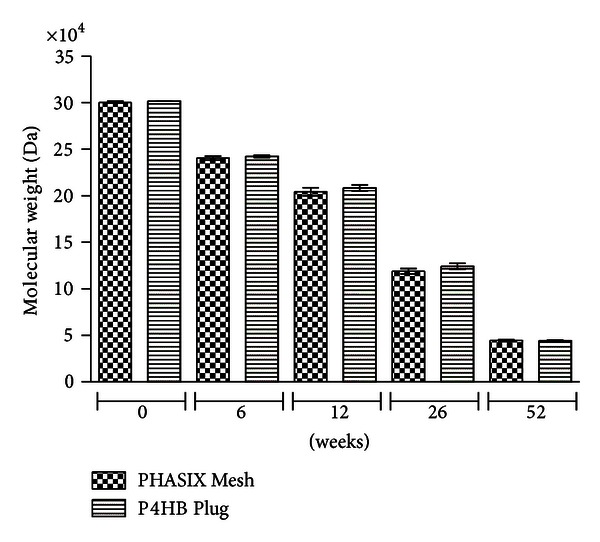
Gel permeation chromatography results showing material resorption of PHASIX Mesh and P4HB Plug increasing significantly over time as evidenced by lower molecular weight over time.

As shown in Figure 5, PHASIX Mesh and P4HB Plug repairs demonstrated similar molecular weights at 0, 6, 12, 26, and 52 weeks with no significant differences detected between the two devices at any of the time points evaluated (*P* = 0.804, *P* = 0.640, *P* = 0.268, *P* = 0.150, and *P* = 0.936, resp.).

The molecular weight of the PHASIX Mesh decreased significantly over time with molecular weights of 240, 510 ± 2, 018 Da, 204, 282 ± 4, 457 Da, 118, 884 ± 2, 821 Da, and 44, 434 ± 879 Da at 6, 12, 26, and 52 weeks, respectively. Molecular weight decreased significantly at each time point compared to the preimplantation (i.e., 0 weeks) molecular weight of the PHASIX Mesh (300, 397 ± 972 Da) with *P* < 0.001 in all cases. Furthermore, the molecular weight of the PHASIX Mesh decreased progressively between 0 and 6 weeks, between 6 and 12 weeks, between 12 and 26 weeks, between 26 and 52 weeks, and overall between 0 and 52 weeks with *P* < 0.001 in all cases.

Similarly, the molecular weight of the P4HB Plug decreased significantly over time with molecular weights of 242, 190 ± 1, 259 Da, 208, 288 ± 3, 420 Da, 124, 126 ± 3, 219 Da, and 44, 148 ± 644 Da at 6, 12, 26, and 52 weeks, respectively. Molecular weight decreased significantly at each time point compared to the preimplantation (i.e., 0 weeks) molecular weight of the P4HB Plug (301, 543 ± 110 Da) with *P* < 0.001 in all cases. Furthermore, the molecular weight of the P4HB Plug decreased progressively between 0 and 6 weeks, between 6 and 12 weeks, between 12 and 26 weeks, between 26 and 52 weeks, and overall between 0 and 52 weeks with *P* < 0.001 in all cases.

#### 3.2.6. Histology

As shown in Table 2, median scores for inflammation were reported in the range of 2-3 for both the PHASIX Mesh and the P4HB Plug at the early 6- and 12-week time points, and both consistently scored 2 at the later 26- and 52-week time points. No significant differences in inflammation scores were detected over time for either the PHASIX Mesh or the P4HB Plug, and no significant differences in inflammation scores were detected between the mesh and plug designs at any of the time points (*P* > 0.05 in all cases).

Macrophage scores of 2 were consistently reported for the PHASIX Mesh at all time points with no significant changes over time (*P* > 0.05). Interestingly, macrophage scores for the P4HB Plug varied slightly over time with a median score of 3 at the 6-week time point, trending toward a significantly lower score of 1 at the 52-week time point (*P* < 0.05). Again, no significant differences were identified between the mesh and the plug designs at any of the time points evaluated (*P* > 0.05 in all cases).

Giant cell scores of 1 were consistently observed for both the PHASIX Mesh and the P4HB Plug at all of the time points. No significant differences in giant cell scores were detected over time for either the PHASIX Mesh or the P4HB Plug, and no significant differences in giant cells scores were detected between the mesh and plug designs at any of the time points (*P* > 0.05 in all cases).

Fibrosis/encapsulation scores for the PHASIX Mesh varied slightly over time with a score of 4 at the 6-week time point that trended toward a significantly lower fibrosis/encapsulation score of 2 at the 12- and 52- week time points (*P* < 0.05). No significant changes in fibrosis/encapsulation scores were observed for the P4HB Plug with scores of 2-3 observed at all time points. In addition, no significant differences in fibrosis/encapsulation scores were detected between the mesh and plug designs at any of the time points.

Granulation tissue/vascularization scores ranged from 2-3 for both the PHASIX Mesh and the P4HB Plug at all time points evaluated. The PHASIX Mesh scores alternated between 3 and 2 at 6 and 12 weeks and again at 26 and 52 weeks, respectively, while the P4HB Plug scores were 2 at the early 6- and 12-week time points and 3 at the later 26- and 52-week time points. However, no significant differences in granulation tissue/vascularization scores were detected over time for either the PHASIX Mesh or the P4HB Plug, and no significant differences were detected between the mesh and plug designs at any of the time points (*P* > 0.05 in all cases).

Collagen morphology scores of 2 were consistently reported for the PHASIX Mesh at all time points with no significant changes over time (*P* > 0.05). Similarly, collagen morphology scores for the P4HB Plug were 1 at the early 6-week time point and 2 at all subsequent time points. As with the mesh design, no significant changes in collagen morphology were detected over time for the plug design (*P* > 0.05), and no significant differences were detected between the mesh and plug designs at any of the time points (*P* > 0.05 in all cases).

## 4. Discussion

Poly-4-hydroxybutyrate (P4HB) is a naturally derived, fully absorbable polymer produced by bacteria via transgenic fermentation techniques. This material has been manufactured into a number of configurations including the PHASIX Mesh and P4HB Plug, which are designed for soft tissue repair applications such as hernia repair. Over the last decade, P4HB materials have been evaluated in a number of animal studies, particularly those investigating cardiovascular applications such as tissue engineered trileaflet heart valves [24], artery augmentation patches [25], and small diameter vascular grafts [26], as well as development of P4HB as a suture material [27]. However, the current study is the first of its kind to evaluate this material specifically for hernia repair applications. The objective of this study was to determine the mechanical properties, resorption profile, and histological characteristics of PHASIX Mesh and P4HB Plug designs when utilized to bridge a surgical defect in the porcine abdominal wall over a period of 6, 12, 26, or 52 weeks. To accomplish this, abdominal wall sites repaired with PHASIX Mesh and P4HB Plug materials were harvested at the end of each implantation period and subjected to mechanical testing, gel permeation chromatography, and histological analysis to assess the strength of the repair, the amount of P4HB material remaining at the repair site, and the host response to the P4HB material, respectively.

Mechanical testing revealed that the burst strength and relative stiffness of the PHASIX Mesh and P4HB Plug repaired sites were similar to each other at all four time points evaluated with no significant differences detected between the two devices for either of these parameters throughout the course of the study (*P* > 0.05 in all cases). In addition, the sites repaired with PHASIX Mesh and P4HB Plug materials also exhibited significantly greater burst strength and relative stiffness compared to the native abdominal wall at all time points regardless of which device was utilized to bridge the defect (*P* < 0.05 in all cases). These results indicate that the P4HB material is capable of augmenting the strength of the native porcine abdominal wall regardless of its configuration as a mesh or plug design. It should also be noted that burst strength and relative stiffness of the PHASIX Mesh and P4HB Plug repaired sites remained stable and did not change significantly between 6 and 52 weeks, suggesting that scaffolds derived from the P4HB material are durable and capable of maintaining support at the repair site over a 52-week period in a porcine model.

Gel permeation chromatography was conducted to quantify the molecular weight of the P4HB material remaining at the repair site at each time point. The data showed that PHASIX Mesh and P4HB Plug possessed similar molecular weights at all time points with no significant differences detected between the two devices at any time (*P* > 0.05 in all cases). These results correspond with the mechanical testing data and demonstrate that the configuration of the P4HB material does not significantly impact its resorption profile. In addition, the molecular weight of both the PHASIX Mesh and the P4HB Plug decreased significantly at each time point compared to the corresponding preimplantation (i.e., 0 weeks) value and also decreased progressively between each time point (i.e., between 0 and 6 weeks, between 6 and 12 weeks, between 12 and 26 weeks, between 26 and 52 weeks, and overall between 0 and 52 weeks). These results indicate that the P4HB material was significantly resorbed over time, but as indicated by the mechanical testing data, significant material resorption did not correspond with a significant drop in mechanical strength at the repair site for either the PHASIX Mesh or the P4HB Plug design.

In addition to mechanical testing and gel permeation chromatography, histological analyses were also performed in order to assess the host response to the P4HB material comprising the PHASIX Mesh and the P4HB Plug designs. No statistically significant changes were observed over time for either the PHASIX Mesh or the P4HB Plug with respect to histological parameters such as inflammation, granulation tissue/vascularization, giant cells, or collagen morphology. Inflammation scores and granulation tissue/vascularization scores ranged from 2-3 for both the mesh and the plug design indicating a mild/slightly detectable to moderate/easily detectable inflammatory response and presence of granulation tissue and vascularization for both devices at all time points. In addition, giant cells scores were consistently reported as 1, indicating a minimal/barely detectable presence of giant cells for both devices at all time points. Similarly, collagen morphology scores were consistently reported as 2, indicating a mixture of both mature (type I, red and orange-red) and immature (type III, green and yellow-green) collagen at all time points.

It should be noted that a few differences were observed with respect to time of implantation. For instance fibrosis/encapsulation scores for the PHASIX Mesh varied slightly over time with a score of 4 at the 6-week time point that trended toward a significantly lower fibrosis/encapsulation score of 2 at the 12- and 52- week time points (*P* < 0.05). These scores correspond to marked/very evident fibrosis/encapsulation at the 6-week time point that was ultimately improved to mild/slightly detectable fibrosis/encapsulation at the later time points. Some degree of fibrosis/encapsulation is expected in the presence of an implanted material such as the PHASIX Mesh, and this response is expected to vary over time with the tissue remodeling/wound healing process. Similarly, the macrophage scores for the P4HB Plug varied slightly over time with a score of 3 at the 6-week time point, trending toward a significantly lower score of 1 at the 52-week time point (*P* < 0.05). These scores correspond to moderate/easily detectable presence of macrophages at the 6-week time point that ultimately improved to only minimal/barely detectable presence of macrophages at the 52-week time point.

In general, no statistically significant differences were detected between the histological scores reported for the PHASIX Mesh design versus the P4HB Plug design for any of the parameters evaluated. These results suggest that the host tissue response was most influenced by the chemical composition of the device (i.e., poly-4-hydroxybutyrate) rather than the structure of the device (i.e., mesh versus plug). Inflammatory infiltrates associated with both devices were predominantly mononuclear with an overall mild response commonly associated with the implantation of a foreign material. Similarly, both devices resulted in comparable, mild to moderate granulation tissue/vascularization that is expected in association with wound healing and tissue remodeling, suggesting that the P4HB material performed appropriately in terms of overall foreign body response regardless of its configuration as a mesh or a plug.

## 5. Conclusions

PHASIX Mesh and P4HB Plug provided durable scaffolds for soft tissue repair in a porcine model. Both repairs demonstrated significant mechanical strength compared to native abdominal wall over a 52-week period, which remained elevated despite significant material resorption over time with no evidence of herniation and/or diastasis. In addition, histological assessment revealed a comparable and mild inflammatory response and mild to moderate granulation tissue/vascularization associated with the P4HB material regardless of its configuration as a mesh or a plug.

## Figures and Tables

**Table 1:** Description of composition, degradation mechanism, and expected human resorption time of absorbable mesh materials.

Mesh type		Composition	Degradation mechanism	Resorption time
DEXON	Covidien (Mansfield, MA)	Polyglycolic acid (PGA)	Hydrolysis	2-3 months
VICRYL	Ethicon, Inc. (Somerville, NJ)	Copolymer of glycolide and lactide	Hydrolysis	2-3 months
GORE BIO-A	W.L. Gore & Assoc., Inc. (Flagstaff, AZ)	Copolymer of poly(glycolide: trimethylene carbonate)	Hydrolysis and enzymatic mechanisms	6 months
TIGR Matrix	Novus Scientific (Uppsala, Sweden)	Comprised of 2 fibers with differing composition and resorption time		
Fiber no. 1		Copolymer of polyglycolide, polylactide, and polytrimethylene carbonate	Hydrolysis	4 months
Fiber no. 2		Copolymer of polylactide and polytrimethylene carbonate	Hydrolysis	36 months
PHASIX	C. R. Bard, Inc./Davol Inc. (Warwick, RI)	Poly-4-hydroxybutyrate (P4HB)	Hydrolysis and enzymatic mechanisms	12–18 months

**Table 2:** Histology scores where 0 = absent/no response, 1 = minimal/barely detectable, 2 = mild/slightly detectable, 3 = moderate/easily detectable, and 4 = marked/very evident for inflammation, macrophages, giant cells, fibrosis/encapsulation, and granulation/vascularization and where 1 = predominantly green (type III collagen), 2 = mixture of green, yellow, and yellow-orange (mixture of type III and type I collagen), and 3 = predominantly red-orange (type I collagen) for collagen morphology.

Histology scores		6 weeks		12 weeks		26 weeks		52 weeks	
		Mean ± SEM	Median	Mean ± SEM	Median	Mean ± SEM	median	Mean ± SEM	Median
Inflammation	PHASIX Mesh	2.6 ± 0.4	2.0	2.4 ± 0.4	3.0	2.2 ± 0.2	2.0	2.0 ± 0.0	2.0
	P4HB Plug	2.8 ± 0.4	3.0	2.0 ± 0.3	2.0	2.4 ± 0.2	2.0	1.6 ± 0.2	2.0

Macrophages	PHASIX Mesh	2.0 ± 0.3	2.0	2.0 ± 0.3	2.0	2.0 ± 0.0	2.0	1.6 ± 0.2	2.0
	P4HB Plug	2.8 ± 0.4	3.0	1.6 ± 0.4	1.0	2.0 ± 0.4	2.0	1.0 ± 0.0	1.0

Giant cells	PHASIX Mesh	1.2 ± 0.2	1.0	1.4 ± 0.2	1.0	1.2 ± 0.2	1.0	1.0 ± 0.0	1.0
	P4HB Plug	1.6 ± 0.4	1.0	1.0 ± 0.0	1.0	1.4 ± 0.2	1.0	1.0 ± 0.0	1.0

Fibrosis/ encapsulation	PHASIX Mesh	3.8 ± 0.2	4.0	2.2 ± 0.2	2.0	2.6 ± 0.2	3.0	2.2 ± 0.2	2.0
	P4HB Plug	3.4 ± 0.2	3.0	2.6 ± 0.2	3.0	2.4 ± 0.2	2.0	3.0 ± 0.0	3.0

Granulation/ vascularization	PHASIX Mesh	2.8 ± 0.2	3.0	2.4 ± 0.2	2.0	2.6 ± 0.2	3.0	2.2 ± 0.2	2.0
	P4HB Plug	2.4 ± 0.2	2.0	2.0 ± 0.3	2.0	2.6 ± 0.2	3.0	2.6 ± 0.2	3.0

Collagen morphology	PHASIX Mesh	1.8 ± 0.2	2.0	2.4 ± 0.2	2.0	2.2 ± 0.2	2.0	2.4 ± 0.2	2.0
	P4HB Plug	1.2 ± 0.2	1.0	2.4 ± 0.2	2.0	2.2 ± 0.2	2.0	2.2 ± 0.2	2.0
